# Immune dysregulation in cancer patients developing immune-related adverse events

**DOI:** 10.1038/s41416-018-0155-1

**Published:** 2018-10-31

**Authors:** Shaheen Khan, Saad A. Khan, Xin Luo, Farjana J. Fattah, Jessica Saltarski, Yvonne Gloria-McCutchen, Rong Lu, Yang Xie, Quan Li, Edward Wakeland, David E. Gerber

**Affiliations:** 1 Department of Immunology, University of Texas Southwestern Medical Center, Dallas, TX 75390-9093 USA; 2 Department of Internal Medicine, University of Texas Southwestern Medical Center, Dallas, TX 75390-9093 USA; 3 Harold C. Simmons Comprehensive Cancer Center, University of Texas Southwestern Medical Center, Dallas, TX 75390-9093 USA; 4 Department of Bioinformatics, University of Texas Southwestern Medical Center, Dallas, TX 75390-9093 USA; 5 Department of Clinical Sciences, University of Texas Southwestern Medical Center, Dallas, TX 75390-9093 USA

**Keywords:** Cancer, Immunology

## Abstract

**Background:**

Up to 40% of cancer patients on immune checkpoint inhibitors develop clinically significant immune-related adverse events (irAEs). The role of host immune status and function in predisposing patients to the development of irAEs remains unknown.

**Methods:**

Sera from 65 patients receiving immune checkpoint inhibitors and 13 healthy controls were evaluated for 40 cytokines at pre-treatment, after 2–3 weeks and after 6 weeks and analysed for correlation with the development of irAEs.

**Results:**

Of the 65 cancer patients enrolled, 55% were women; the mean age was 65 years and 98% received anti-PD1/PDL1 therapy. irAEs occurred in 35% of cases. Among healthy controls, cytokine levels were stable over time and lower than those in cancer patients at baseline. Significant increases in CXCL9, CXCL10, CXCL11 and CXCL13 occurred 2 weeks post treatment, and in CXCL9, CXCL10, CXCL11, CXCL13, IL-10 and CCL26 at 6 weeks post treatment. Patients who developed irAEs had lower levels of CXCL9, CXCL10, CXCL11 and CXCL19 at baseline and exhibited greater increases in CXCL9 and CXCL10 levels at post treatment compared to patients without irAEs.

**Conclusions:**

Patients who developed irAEs have lower baseline levels and greater post-treatment increases in multiple cytokine levels, suggesting that underlying immune dysregulation may be associated with heightened risk for irAEs.

## Introduction

Cancer immunotherapy has revolutionised the treatment of multiple malignancies, with approvals in melanoma, lung cancer, bladder cancer, head and neck cancer, lymphoma, kidney cancer, Merkel cell tumours and microsatellite instability-high cancers. While these promising drugs have led to improved outcomes for thousands of patients, they have also introduced new safety concerns. Immune-related adverse events (irAEs) may affect almost every organ system.^1^ In some cases, these autoimmune toxicities may be severe or even permanent. They may also necessitate treatment interruption and prolonged administration of high-dose steroids and other immunosuppressive agents. Furthermore, immune-related adverse events may occur at any point throughout treatment.^2^

Recent studies indicate that up to 80% of individuals receiving checkpoint inhibitors experience some form of irAE. Approximately 35% of all patients require systemic corticosteroid treatments to mitigate these events. Up to 20% of patients discontinue immunotherapy due to irAEs.^3^ These adverse responses convey substantial morbidity, incur considerable costs and in some cases may preclude further use of these drugs. As immunotherapy use has expanded from major centres to smaller, isolated and less-experienced sites, the ability to recognise and treat immune-related adverse events promptly may be challenged. Further increasing the risk of these toxicities is the emergence of combination immunotherapy regimens, for which the prevalence and severity of immune-related adverse events exceeds those of monotherapy treatments.^4,5^

To date, research into immunotherapy biomarkers has focused largely on the prediction of efficacy. These efforts have almost exclusively investigated tumour characteristics, such as programmed death ligand 1 (PDL1) expression, mutational burden and mismatch-repair deficiency.^6,7^ Thus far, little is known about who is at risk for autoimmune toxicities or when they will occur, concerns that seem more likely related to host immune function than to tumour features. Indeed, the only established approach to limiting immune-related adverse events has been the near-universal exclusion of patients with autoimmune disease from cancer immunotherapy clinical trials, a practice that may impact a substantial proportion of cancer populations.^8^ In the present study, we assessed immune function by analysing levels of 40 cytokines/chemokines and correlated these data with development of irAEs.

## Materials and methods

### Subject recruitment

This study was approved by the UT Southwestern Institutional Review Board (IRB #STU 082015-053). All patients provided informed consent. We enrolled patients with cancer receiving immune checkpoint inhibitor therapy, as well as healthy controls. Potential subjects were identified, screened and recruited through the following mechanisms: (1) direct contact between study team members and clinic personnel to remind staff of the study availability, answer questions and solicit support through patient referrals; (2) systemic data extraction of new immune checkpoint inhibitor orders through the ClinDen Data Extraction system and (3) weekly notifications through the EPIC electronic medical record, through which all referrals for new immunotherapy orders were routed to the study team. Key eligibility criteria included age ≥ 18 years; no prior treatment with immune checkpoint inhibitor-based therapy (PD1, PDL1 and CTLA4 inhibitors); planned for but not yet initiated immune checkpoint inhibitor-based therapy; and willingness to provide the required blood samples and clinical follow-up. For enrolled subjects, the following data were captured: age; sex; race/ethnicity; cancer type and stage; type and dates of immune checkpoint inhibitor therapy and prior therapies.

### Data and sample collection

Peripheral blood samples were collected from patients receiving the checkpoint inhibitor at baseline, after one treatment cycle (either 2 weeks or 3 weeks), and at 6 weeks. Two samples were collected from healthy controls ~2–3 weeks apart. Blood samples were centrifuged at 3000 rpm at 4 °C for 15 min to obtain plasma. Research coordinators collected clinical data, including demographics, cancer type, treatment type and irAEs. A review of all cases for the presence, timing and type of irAE was performed independently by two clinician co-authors experienced in the administration of immune checkpoint inhibitor therapy (S.A.K. and D.E.G.). Any differences between these assessments were discussed with and adjudicated by the entire study team.

### Cytokine/chemokine analysis

Monitoring of cytokine and chemokine levels was performed using Bio-Plex Pro Human Chemokine 40-plex Panel (Bio-Rad Laboratories, Hercules, California) according to the manufacturer’s instructions using a Luminex 200 System. The list of cytokines and chemokines are provided in Supplemental Table 1. Bio-Plex Manager™ 6.1 software was used for data analysis. Concentrations of cytokines and chemokines (pg/mL) were determined on the basis of the fit of a standard curve for mean fluorescence intensity versus pg/mL.

### Statistical analysis

The Mann–Whitney *U* test was used to compare baseline versus post-treatment levels of cytokines/chemokines. The results were expressed as the mean ± standard error (SE). For toxicity analyses, because irAEs may occur throughout the course of treatment with immune checkpoint inhibitors, patients were considered not to have developed irAEs only if they had been followed without evidence of toxicity for at least 6 months. Statistical analyses were performed using GraphPad Prism Software (GraphPad Software Inc, La Jolla, CA, USA). For all statistical analysis, the level of significance was set at *P* < 0.05.

## Results

### Patients

We enrolled a total of 78 subjects, including 65 patients with cancer receiving immune checkpoint inhibitor therapy and 13 healthy controls. Baseline demographic, tumour and treatment data are listed in Table 1. Sixty-four patients received anti-PD1/PDL1 therapy alone (n = 59) or in combination with anti-CTLA4 therapy (*n* = 5). Only one patient in our cohort received anti-CTLA4 monotherapy. Among the 65 patients receiving immune checkpoint inhibitor therapy, we collected baseline samples in 47 cases, samples at 2–3 weeks in 38 cases and samples at 6 weeks in 40 cases. For all 13 healthy controls, we collected paired samples 2–3 weeks apart.

**Table 1:** Characteristics of patients, treatments and irAEs

	No. of patients
*Type of cancer*	
Lung	53
Kidney	5
Melanoma	4
Head/neck	1
Liver	1
Bladder	1
*Treatment*	
PD1	49
PDL1	10
CTLA4	1
PD1 + CTLA4	5
*Immune toxicity*	
Pneumonitis	11
Arthritis	2
Dermatitis	2
Hypophysitis	2
Thyroid	3
Neuro (encephalitis)	1
Complex^a^	3

^a^One case each with the following: thyroid dysfunction and pneumonitis; thyroid dysfunction, dermatitis and nephritis; and hypophysitis and type 1 diabetes

Overall, irAEs occurred in 34% of patients treated with anti-PD1/PDL1 therapy and in 60% of patients treated with a combination of anti-PD1/PDL1 and anti-CTLA4 therapy. The single patient treated with anti-CTLA4 monotherapy developed an irAE.

### Cytokine/chemokine levels

**Fig. 1:**
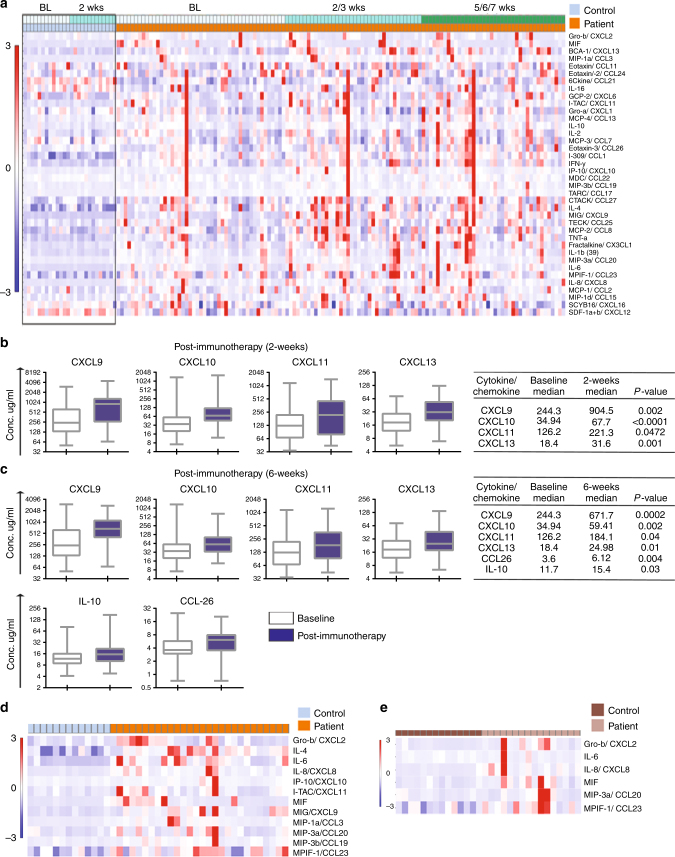
**a** Heatmap showing a comparison of 38 cytokines/chemokines in 13 healthy controls (2-weeks apart) and 47 cancer patients (BL, 2/3 weeks and 5/6/7 weeks post immunotherapy). **b** Serum concentration of CXCL9, CXCL10, CXCL11 and CXCL13 in patients (*n* = 47) at baseline (white boxes) and at 2 weeks post immunotherapy (blue boxes). **c** Serum concentration of CXCL9, CXCL10, CXCL11 and CXCL13 in patients (*n* = 47) at baseline (white boxes) and at 6 weeks post immunotherapy (blue boxes). All changes are statistically significant (*P* < 0.05). The median of each group and *P*-value was calculated using the Mann–Whitney *U* test. **d** Heatmap of significantly upregulated cytokines and chemokines at baseline in cancer patients who did not develop toxicity compared to healthy controls (*P* < 0.05). **e** Heatmap of significantly upregulated cytokines and chemokines in cancer patients at baseline who developed toxicity versus healthy controls (*P* < 0.05)

Among the 40 cytokines/chemokines assessed, the levels of two cytokines (GM-CSF and CXCL5) were below detection levels and were therefore eliminated from analysis. Hierarchical clustering of 38 cytokines and chemokines clearly separated healthy controls from cancer patients, as shown in the heatmap in Fig. 1a. Specifically, 14 cytokines were significantly upregulated in cancer patients at baseline compared to healthy controls. Cytokine levels were stable in healthy controls with no significant differences between the two time points. However, we observed significant changes in cytokine levels in patients after initiation of immunotherapy. At the 2-week time point, there were significant increases in serum levels of CXCL9, CXCL10, CXCL11 and CXCL13 (Fig. 1b). At 6 weeks post treatment, serum levels of CXCL9, CXCL10, CXCL11, CXCL13, IL-10 and CCL26 were significantly upregulated in the immunotherapy patient cohort (Fig. 1c).

Among a total of 47 patients profiled for cytokines/chemokines, 16 patients developed irAEs on checkpoint inhibitor therapy. These autoimmune toxicities included pneumonitis, endocrinopathies, dermatitis, arthritis and encephalitis (Table 1). In the no-irAE group (*n* = 31), at baseline, 12 cytokines were significantly upregulated when compared to healthy controls (Fig. 1d). The irAE group (*n* = 16) had significantly elevated levels of five cytokines (IL-6, CXCL2, CCL20, CXCL8 and CCL23) compared to healthy controls (Fig. 1e).

**Fig. 2:**
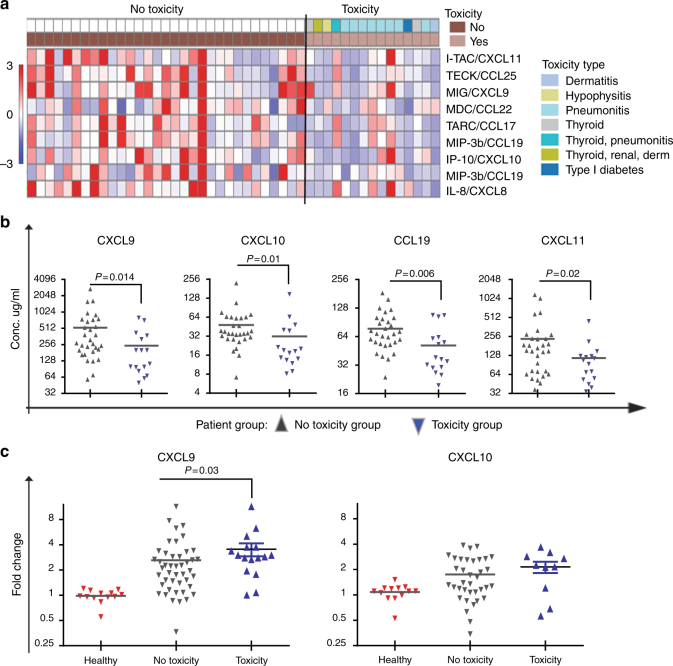
**a** Heatmap of significantly downregulated cytokines and chemokines in cancer patients who did not develop toxicity (no-toxicity group coloured in dark brown) versus patients who developed toxicity (toxicity group coloured in light brown) (*P* < 0.05). **b** Comparison of serum concentration of CXCL9, CXCL10, CXCL11 and CCL19 between two groups of patients at baseline before the initiation of immune checkpoint blockade: the toxicity group in grey and no-toxicity group in blue. All changes are statistically significant (*P* < 0.05). The median of each group and *P*-value was calculated using the Mann–Whitney *U* test. **c** Fold change in serum levels of CXCL9 and CXCL10 at 6 weeks post treatment compared to baseline is calculated between the toxicity group (grey) and no-toxicity group (blue). The median of each group and *P*-value was calculated using the Mann–Whitney *U* test (*P* < 0.05)

Notably, patients who developed irAEs had lower baseline serum levels of several cytokines/chemokines compared to patients who did not develop irAEs (Fig. 2a). In particular, significantly lower levels of CXCL9, CXCL10, CXCL11 and CCL19 at baseline were observed in the irAE group (Fig. 2b). Conversely, the fold increase in cytokines/chemokines at 2–3 weeks and at 6 weeks (particularly for CXCL9 and CXCL10) was significantly greater in the irAE group (Fig. 2c).

We also performed these analyses restricting to cases that received only anti-PD1/PDL1 therapy without anti-CTLA4 therapy (42 patients; 12 patients with irAEs). There was no meaningful change to the results.

## Discussion

Despite extensive preclinical research, several drug approvals in multiple cancer types and hundreds of ongoing clinical trials in cancer immunotherapy, irAEs remain a largely understudied and poorly understood phenomenon. Complicating the study of these autoimmune toxicities is their diverse and unpredictable presentation. To date, apart from the hypothetical concern that a pre-existing autoimmune disease may be exacerbated by immune checkpoint inhibitor therapy (resulting in exclusion of these patients from most immunotherapy clinical trials), there have been no clear demographic or clinical factors associated with these events.^8,9^ Furthermore, in contrast to classic toxicities of conventional chemotherapy such as myelosuppression, irAEs may occur at any point in therapy and rarely have a discrete laboratory test to aid in diagnosis. Recognising that irAEs are more likely to reflect host rather than tumour biology, in this study we focused on markers of systemic immune status. We found that patients with lower levels of pre-treatment immune markers experienced greater increases in these parameters after treatment initiation, and also had a greater risk of irAEs.

These findings are reminiscent of earlier observations that autoimmune diseases may occur at greater rates in populations with immune dysregulation. A classic example is the association of HIV/AIDS with autoimmunity.^10^ In this population, the frequency of reported rheumatologic conditions ranges up to 60%. Specific reported diseases include systemic lupus erythematosus, anti-phospholipid syndrome, vasculitis, immune thrombocytopenic purpura, polymyositis and others. Coupled with these epidemiologic findings, our current study suggests that immune dysregulation may heighten the risk of autoimmune events, specifically irAEs in patients treated with immune checkpoint inhibitors. Notably, autoimmune diseases are mostly likely to manifest in HIV/AIDS populations during periods of immune reconstitution,^10^ which may be analogous to the period after initiation of immune checkpoint inhibitors in cancer populations.

In the present study, patterns of inducible CXCL 9, 10, 11 and 13 levels had the strongest association with irAEs. All were lower at baseline, suggesting that this measurement may be of predictive value for individuals at risk to develop irAEs. Furthermore, CXCL 9 and 10 had particularly large increases after therapy started. These interferon gamma-inducible small cytokines (CXCL 9/10/11) bind the chemokine receptor CXCR3 and are chemotactic for activated T cells. They have been implicated in a variety of autoimmune conditions, including thyroiditis, type 1 diabetes mellitus, Addison’s disease, inflammatory bowel disease and systemic sclerosis.^11,12^ CXCL 13, a chemokine that binds CXCR5, is expressed on mature B cells, CD4+ follicular helper T cells (Tfh) and activated tonsil T regulatory cells (Tregs).^13,14^ CXCL13 functions as a B-cell chemoattractant and is involved in multiple autoimmune and inflammatory diseases, including multiple sclerosis, rheumatoid arthritis, Hashimoto’s thyroiditis, Sjogren’s syndrome, systemic lupus erythematosus and myasthenia gravis.^15–18^

In addition to autoimmune disease, the CXCL9/10/11–CXCR3 axis has been a major focus of research in the tumour microenvironment (TME)^19^. It regulates the differentiation of naïve T cells to T helper 1 (Th1) cells and leads to migration of immune cells to tumour sites. Studies have shown that CXCR3 expression on T cells/CXCL9–10 expression in tumour tissue is associated with increased tumour-infiltrating lymphocytes (TILs) accumulation and a favourable clinical outcome.^20–22^ The axis is also implicated in PDL1/PD1 therapy, whereby anti-PD1 therapy enhances T-cell-mediated tumour regression by increasing the expression of interferon gamma (IFNγ)-inducible chemokines.^23,24^ To our knowledge, this is the first study to report the level of these key immune-regulatory chemokines correlating with the development of irAEs and highlighting their role in the development of autoimmune toxicities on checkpoint inhibitor therapy. Our findings implicate the potential of these cytokines as biomarkers in predicting the development of irAEs. That these cytokines may be involved in both autoimmune events and antitumour effects may in part explain why some studies have shown that irAEs are associated with better treatment outcome from checkpoint inhibitors.^25^

Cytokine levels have been evaluated in earlier studies of immune checkpoint inhibitors, with primary focus on antitumour efficacy.^26^ One study of preoperative ipilimumab for melanoma found that baseline interleukin-17 was associated with the development of diarrhoea/colitis.^27^ That we identified a different pattern of cytokines in the present study may reflect the predominance of anti-PD1/PDL1 therapy and low rates of gastrointestinal toxicity (which is more commonly associated with anti-CTLA4 treatment).

Our observation of baseline-elevated cytokine/chemokine levels among individuals with cancer compared to healthy controls is consistent with findings from earlier studies. In colorectal cancer, serum cytokine alterations occur in a stage-dependent fashion.^28^ In pancreatic cancer, levels of IL-6, -8, -10 and TNFα are higher than in healthy controls.^29^ Whether these profiles represent a chronic inflammatory state predisposing to cancer, or a reaction to the malignancy, remains unclear. Future studies will need to focus on understanding the association of cytokine levels with immune regulation status. Whether these patients have differences in the number/function of exhausted T cells, regulatory T cells or T-cell receptor repertoire remains unknown.

While research into irAEs remains a nascent field, for some conditions, predictive biomarkers are emerging. Among patients treated with pembrolizumab, anti-thyroid antibodies were present in 80% of patients who developed thyroid dysfunction, compared with 8% of patients who did not (*P* < 0.0001).^30^ Although this observation suggests that PD1 blockade modulates humoral immunity, it is less likely that discrete and tissue-specific autoantibodies will be identified for most irAEs. Indeed, many autoimmune disorders, particularly those involving the lung, tend not to have serologic correlates.

Limitations of this study include the single-centre setting, the predominance of a single cancer type (lung cancer), the paucity of anti-CTLA4 cases and the inherent challenges of clinically diagnosing and characterising many irAEs. Relatively small patient numbers preclude the analysis according to type of immunotherapy, or type and severity of irAE. Strengths include the availability of serial specimens, the requirement that patients have at least 6 months of follow-up before being considered without irAE, the use of rigorous statistical methods to account for repeated testing, the inclusion of large numbers of patients exposed to anti-PD1/PDL1 therapies and the confirmation of our findings in an exclusively anti-PD1/PDL1 population. Indeed, despite broad approval of anti-PD1/PDL1 therapies across cancer types, to date, relatively few studies of systemic biomarkers have focused on this population. This ongoing prospective study has already revealed an important irAE association with specific cytokines/chemokines by performing immune profiling with a comprehensive cytokine/chemokine panel. We are collecting an extensive set of patient serial samples and anticipating vigorous follow-up using several other assays of the patient’s immune system in the future. Finally, our current analysis indicates a potential to build a prediction model to estimate patients’ risk of irAE in the future. The incorporation of data from pre-treatment baseline and after 1–2 doses of checkpoint inhibitor therapy (before the onset of most irAEs) means that these or the related biomarkers could serve to guide patient management in real time.

In conclusion, among cancer patients treated with immune checkpoint inhibitors, irAEs may be more common among those exhibiting immune dysregulation. Specifically, cases with irAEs had lower baseline levels and greater post-treatment increases in cytokines associated with T-cell activation and autoimmune disease. Biomarkers for the prediction and tracking of autoimmune toxicity in this population could serve to customise therapy, tailor monitoring and even expand the use of checkpoint inhibitors to groups in which they are currently avoided.

## Electronic supplementary material

Supplemental Table 1

## Availability of data and material

The datasets generated during and/or analysed during the current study are available from the corresponding author on reasonable request.
