# Supplementary material for: Immune dysregulation in cancer patients developing immune-related adverse events
Source: Br J Cancer. 2018 Oct 31;120(1):63–8. doi: 10.1038/s41416-018-0155-1 (PMC6325132; doi:10.1038/s41416-018-0155-1)
Supplement: Supplementary file 1 — Supplemental Table 1 [file 41416_2018_155_MOESM1_ESM.docx]

**Supplemental Table 1.** Cytokines and chemokines included in the analysis

| **Cytokines and Chemokines** | | |
| --- | --- | --- |
| 6Ckine/ CCL21 | ENA-78/ CXCL5 | IFN-y |
| CTACK/ CCL27 | BCA-1/ CXCL13 | IL-1b |
| Eotaxin/ CCL11 | Fractalkine/ CX3CL1 | IL-2 |
| Eotaxin-2/ CCL24 | GCP-2/ CXCL6 | IL-4 |
| Eotaxin-3/ CCL26 | Gro-a/ CXCL1 | IL-6 |
| I-309/ CCL1 | Gro-b/ CXCL2 | IL-10 |
| MCP-1/ CCL2 | SCYB16/ CXCL16 | IL-16 |
| MCP-2/ CCL8 | SDF-1a+b/ CXCL12 | GM-CSF |
| MCP-3/ CCL7 | IP-10/ CXCL10 | MIF |
| MCP-4/ CCL13 | I-TAC/ CXCL11 |  |
| MDC/ CCL22 | MIG/ CXCL9 |  |
| MIP-1a/ CCL3 | IL-8/ CXCL8 |  |
| MIP-1d/ CCL15 |  |  |
| MIP-3a/ CCL20 |  |  |
| MIP-3b/ CCL19 |  |  |
| MPIF-1/ CCL23 |  |  |
